# Enhanced Micro-Channeling System via Dissolving Microneedle to Improve Transdermal Serum Delivery for Various Clinical Skincare Treatments

**DOI:** 10.3390/pharmaceutics14122804

**Published:** 2022-12-14

**Authors:** Jeeho Sim, SeongDae Gong, Geonwoo Kang, Mingyu Jang, Huisuk Yang, Jaesung Park, Youngchan Kim, Hyunkyu Lee, Hyunji Jung, Youseong Kim, Chansol Jeon, Hyeri Ahn, Minkyung Kim, Jaibyung Choi, Ho Lee, Hyungil Jung

**Affiliations:** 1 Department of Biotechnology, Yonsei University, 50 Yonsei-ro, Seodaemun-gu, Seoul 03722, Republic of Korea; 2 JUVIC Inc., No. 208, Digital-ro 272, Guro-gu, Seoul 08389, Republic of Korea; 3 Graduate School, Department of Mechanical Engineering, Kyungpook National University, 80 Daehak-ro, Buk-gu, Daegu 41566, Republic of Korea; 4 School of Convergence, Department of Robot and Smart System Engineering, Kyungpook National University, 80 Daehak-ro, Buk-gu, Daegu 41566, Republic of Korea; 5 Institute of Nanophotonics Application, Kyungpook National University, 80 Daehak-ro, Buk-gu, Daegu 41566, Republic of Korea; 6 Laser Application Center, Kyungpook National University, 70 Dongnae-ro, Dong-gu, Daegu 41061, Republic of Korea

**Keywords:** serum, topical application, dissolvable micro-channeling system, transdermal delivery, skin hydration, skin depigmentation, wrinkle improvement

## Abstract

Topical liquid formulations, dissolving microneedles (DMNs), and microscale needles composed of biodegradable materials have been widely used for the transdermal delivery of active compounds for skincare. However, transdermal active compound delivery by topical liquid formulation application is inhibited by skin barriers, and the skincare efficacy of DMNs is restricted by the low encapsulation capacity and incomplete insertion. In this study, topical serum application via a dissolvable micro-channeling system (DMCS) was used to enhance serum delivery through micro-channels embedded with DMNs. Transdermal serum delivery was evaluated after the topical-serum-only application and combinatorial serum application by assessing the intensity of allophycocyanin (APC) loaded with the serum in the porcine skin. APC intensity was significantly higher in the skin layer at a depth of 120–270 μm upon combinatorial serum application as compared to topical-serum-only application. In addition, the combinatorial serum application showed significantly improved efficacy in the clinical assessment of skin hydration, depigmentation, improvement of wrinkles, elasticity, dermal density, skin pores, and skin soothing without any safety issues compared to the serum-only application. The results indicate that combinatorial serum application with DMCS is a promising candidate for improving skincare treatments with optimal transdermal delivery of active compounds.

## 1. Introduction

Transdermal delivery of therapeutics or functional substrates, which are widely used for skin-mediated drug treatment, is patient friendly and can be used as an alternative for oral drug delivery, which causes drug degradation in the digestive system and has a low absorption rate [1,2,3,4]. Topical application of formulations, such as ointments and creams, is mostly used for various cosmetic skincare treatments, such as skin hydration, skin depigmentation, and anti-wrinkling [5,6,7]. They are also used for skin-mediated treatment targeting local skin diseases, such as skin melasma, atopic dermatitis, and psoriasis [8,9,10,11]. However, active compounds with high molecular weights have difficulty penetrating the stratum corneum, which acts as a barrier, thereby limiting drug performance to a low level [12]. Therefore, the creation of micro-channels to overcome the stratum corneum barrier for effective delivery of active compounds has been investigated [13,14].

Solid microneedles (SMNs) composed of metal, silicon, and polylactic acid generate microscopic perforations and have been commercialized as products, such as Dermaroller^®^ (Dermaroller Deutschland GmbH, Wolfenbuettel, Germany) [15,16,17,18]. The perforations created by SMNs induced permeation of the applied formulation into the skin, thereby extensively enhancing cosmetic effects [15]. In addition, the minimal epidermal damage created by the SMNs induces a wound healing cascade, which leads to neovascularization and neocollagenesis, enabling the curing of acne scars [18]. However, solid microneedles have several shortcomings that arise from their bio-incompatibility, which leads to the risks of inflammation, infection, irritation, and redness. [19,20]. Moreover, SMNs waste after application is sharp, potentially biohazardous, and can be misused by users, which threatens their safety [21,22].

Therefore, dissolving microneedles (DMNs), drug-encapsulated dissolvable microscale-polymeric needles, were investigated to eliminate the safety issues that emerge after SMNs application. DMNs have been designed to penetrate skin barriers and release drugs into perforated skin channels after dissolution by body fluid in the skin [23,24]. To ensure user safety, DMNs are fabricated using biocompatible and degradable FDA-approved polymers, such as polyvinyl pyrrolidone, carboxymethyl cellulose, and hyaluronic acid, which have been extensively researched for cosmetic and medical treatments [25,26,27,28,29]. Indeed, DMNs encapsulating niacinamide and ascorbic acid significantly increased the delivery of niacinamide to the skin and the antioxidant activity of ascorbic acid in the skin [24]. In addition, the application of DMNs encapsulating adenosine, which is known to improve skin wrinkles, showed improved cosmetic effects compared with the topical application of adenosine cream [25]. However, the low length and small volume of cosmetic DMNs, which are so designed to prevent skin cell damage and inflammation, lead to the encapsulation of limited amounts of functional compounds [30]. Therefore, the skincare effects of functional compounds carried by cosmetic DMNs are limited, and incomplete insertion into the skin hinders the complete delivery of functional compounds [31,32]. In addition, the outbreak of stressful factors, such as heat, ultraviolet, and air-blowing processes during DMNs fabrication procedures, may adversely affect the activity of the drugs encapsulated in the DMNs, which reduces efficacy [33].

Several studies on the combination of DMNs and topical formulations application have been conducted to overcome the limitations of DMNs, such as limited dose encapsulation, incomplete insertion, and activity reduction in encapsulated drugs [34,35]. Sequential applications of topical serum and DMNs were relatively effective compared to traditional topical serum or DMNs application [34]. To prevent the partial dissolution of the DMNs by the topically applied serum immediately before insertion into the skin, an optimal interval (5 min) between topical serum application and DMNs application was suggested. However, users who may not maintain optimal intervals will experience poor channeling performance of DMNs, which leads to reduced efficacy of the serum. Thus, the application of adenosine-loaded topical cream immediately after separating DMNs from the skin for effective delivery of cream through open-state perforation was further investigated, and the effective delivery of adenosine was confirmed [35]. However, channels that were formed shortly after the separation of DMNs due to shrinkage may have a smaller size than those that were formed when DMNs were embedded. Although previous research combining the application of DMNs and topical formulations showed enhanced efficacy, it had the issue of user inconvenience and still had room for further improvement. Therefore, the simultaneous application of DMNs and topical application to deliver serum with DMNs embedded in the skin was investigated to overcome the shortcomings of previous studies.

In this study, a dissolvable micro-channeling system (DMCS) was designed to integrate topical serum application with DMN simultaneously. It consisted of: (i) DMNs fabricated on a polystyrene disk with a central hole to infuse the serum, (ii) groove structures to facilitate even serum spread, and (iii) a dropper for serum infusion. Serum containing niacinamide, adenosine, and Lactobacillus fermented solution (LFS) approved for the effects of skin hydration, depigmentation, wrinkle care, and conditioning was prepared for infusion through the dropper after DMNs insertion into the skin. The serum infused through the central hole of the disk saturates the groove structures and eventually spreads throughout the skin area covered with the disk. The efficacy of transdermal serum delivery was visualized by loading allophycocyanin (APC) with the serum, and a significantly larger amount (*p* < 0.05) of serum labeled with APC was detected in the layers of porcine skin, with 120–270 μm depth. Furthermore, the superior efficacy of transdermal delivery of the active compounds in the serum by combinatorial application of serum and DMCS was shown in a randomized clinical study by assessing skin hydration, area of pigmentation, wrinkle index in human crow’s feet, dermal density, dermal elasticity, density of skin pores, and skin soothing effect. The novel approach of combining DMNs application and topical serum application by DMCS achieved enhanced transdermal delivery and optimal performance of active compounds without apparent safety issues.

## 2. Materials and Methods

### 2.1. Fabrication of DMCS

DMNs were fabricated on a rectangular polystyrene disk with groove structures on every side of the disk in a diagonal position to assist the flow of the serum. First, to increase the hydrogen bond strength between the polymer and disk, hydrophilic plasma treatment was performed using a plasma cleaner (Harrick Plasma, Ithaca, NY, USA). Hyaluronic acid (HA) (average molecular weight 30 kDa, Soliance, Pomacle, France) was dissolved in distilled water to produce 70% (*w/v*) and 40% (*w/v*) HA solutions and homogenized using a paste mixer (KMtech, Gyeonggi, Korea). The solution was dispensed on the disk using a dispenser (Musashi Engineering, Tokyo, Japan) to form 72 droplets and left to dry for 2 h. Then, a homogenized 40% HA solution was laminated onto the dried droplets and fabricated into DMNs by centrifugal lithography [33], exerting a centrifugal force of 320× *g* for 2 min under vacuum conditions. The fabricated DMNs were visualized to analyze their morphological properties using a bright-field microscope (Leica, Wetzlar, Germany).

### 2.2. Mechanical Strength Evaluation of DMNs

To ensure that the DMNs had enough mechanical strength for skin penetration, the fracture force of a single DMN was analyzed using a material testing machine (OmniTest 5.0, Mecmesin Ltd., West Sussex, UK). The DMNs with the disk were placed on the test stage, and a metal probe was moved downward at a continuous speed of 2.0 mm per min. After the probe arrived at the tip of the DMN, the magnitudes of the axial force according to the displacement of the probe were detected and expressed as a graph. The peaks in the graphs, which indicate the fracture forces, were recorded.

### 2.3. In Vitro Skin Insertion Test

DMNs with the disk were inserted into porcine skin (Cronex, Hwaseong, Korea) by applying thumb force for 5 s to evaluate the in vitro skin penetration. After 1 min of application, the disk was removed, and 0.4% trypan blue solution (Sigma-Aldrich, St. Louis, MO, USA) was applied to the insertion site of porcine skin for staining for 30 min. Excess trypan blue was removed, and porcine skin was taped to remove the residual trypan blue solution. Stained skin was analyzed by visualizing the images using a bright-field microscope. In addition, the dissolution rates of the DMNs under serum supply were evaluated based on the change in height of the DMNs. After applying the DMCS, the DMNs group without serum infusion was detached after 1 min. The DMNs group with serum infusion was infused with the serum after 30 s, followed by detachment after 1 min.

### 2.4. Assessment of Transdermal Delivery through DMCS

To visualize the delivery of serum through the micro-channels formed on porcine skin after DMNs application, allophycocyanin (APC) (1%, Sigma Aldrich, St. Louis, MO, USA) was used as an indicator of serum distribution in the porcine skin tissue. In addition, to visualize the dissolved DMNs in the skin, calcein (0.1%, Sigma Aldrich, St. Louis, MO, USA) and rhodamine B (Rho B) (0.1%, Sigma Aldrich, St. Louis, MO, USA) were loaded to serum, base layer of DMNs, and a laminated layer of DMNs, respectively. To assess the transdermal delivery of DMNs, we carried out optical sectioning employing a confocal laser scanning microscope (CLSM). The visualization of drug delivery inside the tissue was conducted for two different groups. The first group is the topical treatment of serum labeled with APC without DMNs. The second group is the topical application of APC in conjunction with DMNs. The serum dose was kept constant at 300 μL, for both conditions. The optically sectioned image was taken at a particular depth and with a time interval (at 0, 30, and 60 min after the application of functional agents). Images were taken every 30 μm depth intervals up to about 330 μm.

### 2.5. CLSM for Non-Invasive Visualization of Transdermal Delivery

Three continuous wave (CW) lasers, with 532 nm (35-KAP-431-220, Melles Griot, Rancho Santa Margarita, CA, USA), 488 nm (iBeam Smart-488S, Toptica, Gräfelfing, Germany), and 635 nm (HNL050R, Thorlabs, Newton, NJ, USA), were used as the light sources of the CLSM for excitation of Rho.B, calcein, and APC fluorescence probe encapsulated in the DMNs and serum. The CLSM used two scanning mirrors for rasterizing the laser beam in two dimensions across the sample stage surface. The first consists of a polygon mirror, which performs the fast axis horizontal line scanning by its rotation. The second is a galvanometer-mounted mirror, which carries out the slow axis vertical line scanning. The scanned beams were focused by an objective lens (10×, NA 0.45, Zeiss, Oberkochen, Germany) onto the sample. Fluorescent lights from the sample were collected by the objective lens, then returned along the beam path until they were partially diverted by a beam splitter toward a dichroic mirror [36].

The fluorescent lights emitted from the sample returned along the same irradiation beam path until splitting at specific positions by a beam splitter. The splitting beam is again separated by wavelength by a dichroic mirror and then entered into the photo-multiplier tube (PMT: R9110, Hamamatsu, Shizuoka, Japan). Each beam that entered the PMT was focused by employing a pinhole and an achromatic lens. The analog signal generated by light collected at the PMT is converted into an 8-bit digital signal using a frame grabber (Solios eA/XA, Matrox Imaging, Montreal, QC, Canada). This signal is organized into images by homemade GUI software. The pinhole serves as a spatial filter to achieve optical sectioning in this case by selectively passing only rays from the focal plane and rejecting out-of-focus ones, making it a crucial component of CLSM. Due to optical sectioning, imaging inside the tissue can be accomplished without invasive procedures. The acquired *en face* image has a 880 × 825 μm (512 × 480 pixels) field of view (FOV).

### 2.6. Randomized Clinical Trial of Serum-Only Application and Combinatorial Application

Twenty healthy women with an average age of 51.35 ± 4.49 who had hyperpigmented areas with wrinkles around their eyes (crow’s feet region) agreed with the purpose of the investigation, acknowledged the precautions, and were selected to participate in the four-week trial. Randomized clinical trials of serum-only application and combinatorial application of DMCS with serum were conducted with institutional review board (IRB) approval from the P&N Skin Research Center (approval number: PNK-22516-M2R, approved on 24 June 2022) in accordance with the ethical rules of the Declaration of Helsinki. A serum-only application was conducted on the total area of the right side of the face, and a combinatorial application was conducted on the crow’s feet area, hyperpigmented area, and the cheek on the left side of the face. Every subject was instructed to apply serum twice daily (morning and evening) and DMCS with serum twice a week (evening). For each subject, a 300 mg dose of serum was applied per usage. Skin hydration, pigmented area, wrinkles, dermal density, elasticity, pore number, pore size, and soothing effects after use were evaluated during the trial.

### 2.7. Skin Hydration Evaluation

The skin hydration retention rate and trans-epidermal water loss were assessed to evaluate skin hydration. Corneometer^®^ CM 825 (Courage+Khazaka Electronic, GmbH, Kern, Germany), which detects skin moisture content by sensing reduced resistance after applying an alternative wave, was used to determine the skin hydration retention before and after use, and 24 and 48 h after use. A vapometer (Delfin Technologies Ltd., Kuopio, Finland), a closed-chamber evaporimeter consisting of two sensors monitoring relative humidity and temperature changes, was used to evaluate the evaporation rate (g/m^2^/h) to determine the trans-epidermal water loss before and after use.

### 2.8. Evaluation of the Depigmenting Effect on Hyperpigmented Spots

To compare the depigmentation effect of the respective serum and combinatorial application, hyperpigmented spots under the eye rims were imaged using Antera 3D CS (Miravex Ltd., Dublin, Ireland), which contains a camera for acquiring skin images by illuminating the skin with LEDs of various wavelengths in an area of 3136 mm^2^ (56 × 56 mm), and a corresponding software tool for analyzing the skin. The skin chromophore concentrations before use and at 2- and 4-week use were derived from the spectral analysis of the acquired image data. The acquired images were analyzed using the melanin hyperconcentration mode to measure the melanin index, which represents the skin pigmentation effect.

### 2.9. Evaluation of Skin Wrinkles through Three-Dimensional Visualization

Three-dimensional visualization of skin wrinkles in the crow’s feet area was performed using Antera 3D CS by constructing a tomography of the skin texture. The maximum depths (mm) of the wrinkles were derived from the saved images, and the reduction in the maximum depth implies an improvement in wrinkles in the crow’s feet region. The same sites at each evaluation time point (before and after use, and at 2- and 4-week use) were visualized during the trial. The skin wrinkle differences between the left and right sides were analyzed to verify the efficacy of the products.

### 2.10. Skin Elasticity Assessment

Cutometer MPA580 (Courage+Khazaka Electronic, GmbH, Germany), which precisely displays skin resilience in a graphical and numerical manner, was used to evaluate skin elasticity. The skin was drawn by the aperture of the probe by consistently exerting a negative pressure of 450 mbar for 2 s and then left with pressure off for 2 s. After three repetitions, the resistance of the skin to pressure and its tendency to return to the original state were measured. The R2 parameter, which indicates the viscoelasticity of the skin, was evaluated before use and at 2- and 4-week use.

### 2.11. Dermal Density Assessment

A diagnostic ultrasound device, Dermascan^®^ C (Cortex Technology, Hadsund, Denmark), which images the sound waves reflected from the adjacent human body, was used to evaluate dermal density. The density of the skin tissue was determined by calculating the reflected sound waves among skin tissues with different densities and was examined using the B-scan model. Two-dimensional images of echo intensity, which indicate the dermal density of the epidermis and dermis, were obtained. Dermal density analysis and images were obtained before use and at 2- and 4-week use.

### 2.12. Skin Pore Number and Area Measurement

Skin pores on both sides of the cheeks were imaged using Antera 3D CS, and the skin topography was derived from the spatial analysis of the acquired image by reconstructing the skin surface in two and three dimensions. The number (ea) of skin pores and total area (mm^2^) of skin pores were derived from the images captured before and after use. The changes in the number and total area of the skin pores were analyzed, and the reduction in each parameter represents an improvement of the skin pores.

### 2.13. Evaluation of Skin Soothing Effect after External Stimulation

Specialty film, D-squame Scan 850A Instrument (Cuderm, Dallas, TX, USA), was used to provide external stimulation to the human facial skin by repeating the attachment and detachment (tape stripping), thereby inducing skin damage. The stimulated skins were treated by three different measures for soothing, and the soothing effects were compared. Antera 3D CS was used to image the skin before stimulation, after stimulation, and after application. The acquired images were then converted into hemoglobin hyperconcentration mode to analyze redness to evaluate the efficacy for skin soothing.

### 2.14. Skin Irritation and Sensitization Assessment

Skin irritation and sensitization of the serum-only application group and combinatorial group were measured after treatment and at 24 h, 48 h, 2 weeks, and 4 weeks after treatment. Objective abnormal skin reactions, including erythema, edema, and scale, were tested by visual evaluation of the experts, and subjective irritations, including itching, tingling, burning, stiffness, and prickling, were assessed by self-observation of the testers.

### 2.15. Statistical Analysis

SPSS^®^ Package (IBM, New York, NY, USA) was used to verify the statistical significance of the measurements after use compared with the measurements before use. Significance was confirmed when the significance probability was <5% (*p* < 0.05) in the 95% confidence interval. Normality was verified using the Shapiro–Wilk method. Comparison of before and after use was performed using the paired *t*-test, which is a parametric method, and by the Wilcoxon signed rank test, which is a non-parametric method. In parametric cases, ANOVA was used to analyze repetitive data three or more times from the same subjects, and a post hoc comparison was performed using the Bonferroni correction. In non-parametric cases, the Friedman test was used for analysis, and paired comparisons were performed using the Wilcoxon signed rank test, followed by revision using the Bonferroni correction method. Comparisons between the two groups at each point were performed by repeated-measures ANOVA when homogeneity was satisfied and by ranked ANCOVA when homogeneity was not achieved. Comparisons between more than three groups at each point were performed by one-way ANOVA, then revised by the Bonferroni correction when homogeneity was satisfied, and by the Kruskal–Wallis test followed by revision with the Mann–Whitney test when homogeneity was not achieved.

## 3. Results and Discussion

### 3.1. Concept of Serum Infusion after DMCS Application

**Figure 1:**
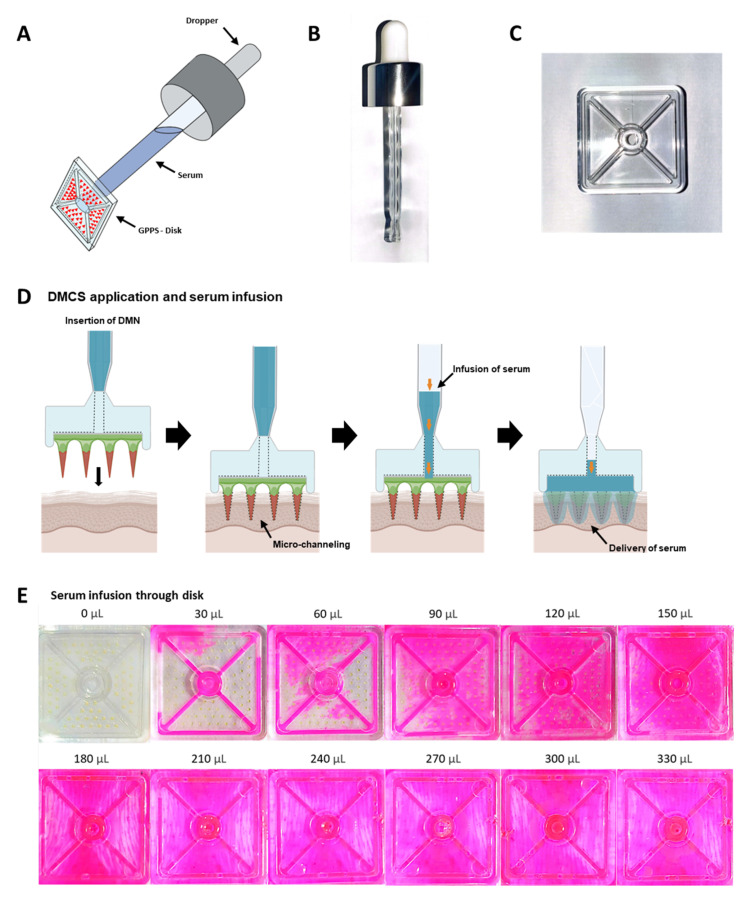
(**A**) Schematic illustration of DMCS consisting of DMNs, disk, and dropper. (**B**) Image of dropper and (**C**) disk. (**D**) Process of serum infusion in the disk. Schematic illustration of serum infusion after DMCS application on the skin. After micro-channels were formed by DMNs penetration, serum infusion was performed, followed by dissolution of DMNs and diffusion of serum into the epidermis. (**E**) Progression of serum infusion in the disk.

As shown in Figure 1A, the DMCS was assembled by joining the end part of the dropper (Figure 1B) to a hole with a diameter of 4 mm in the polystyrene disk (Figure 1C). The delivery of niacinamide, adenosine, and active compounds loaded in the LFS, which are supplied from the serum into the skin, is accomplished by the combinatorial use of DMN and topical serum application, as shown in Figure 1D. When applying DMCS, first, the fabricated DMNs on the disk were primarily inserted into the skin to create micro-channels, followed by serum infusion into the cavity formed between the disk and skin surface. Because the infused serum spread out on the skin surface where the DMNs were inserted, dissolution of the DMNs was induced. The active compounds in the serum are delivered to the intracutaneous region via micro-channels, which are created by DMNs. The combined use of DMN and topical serum overcame the limitations of DMNs and topical application simultaneously. That is, the supply of serum to the micro-channels surmounts the low loading capability of DMN, and the delivery of the active compound via the micro-channel resolves the issue of low delivery efficacy of topical application.

Even distribution of active compounds to the porcine skin surface covered by the disk is essential to deliver a uniform amount of active compounds to all micro-channels on the skin created by the DMNs. Therefore, even serum spread on the disk to contact all DMNs is critical during serum infusion. We designed two diagonal grooves and four side grooves with a depth of 500 μm and width of 1 mm to induce an even spread of serum on the disk when the DMCS was applied to the skin. To evaluate serum spread, DMCS with different Rho B-loaded serum volumes were infused and spread throughout the disk. Sera with volumes of 0, 30, 60, 90, 120, 150, 180, 210, 240, 270, 300, and 330 μL were infused onto porcine skin and imaged (Figure 1E). When 30 μL serum was infused, only diagonal grooves and two grooves on the sides were filled. As the serum volume increased to 60 μL, excess serum was observed outside the grooves. This implied that the infused serum filled the diagonal and side grooves first and then overflowed from the groove. The DMNs on the disk were not fully in contact with the serum when it was infused at less than 120 μL. Therefore, the results indicated that the minimum amount of serum required to fully dissolve the DMNs fabricated on the disk was 180 μL.

### 3.2. Morphology, Skin Penetration, and Serum-Induced Dissolution Analysis of DMNs on DMCS

**Figure 2:**
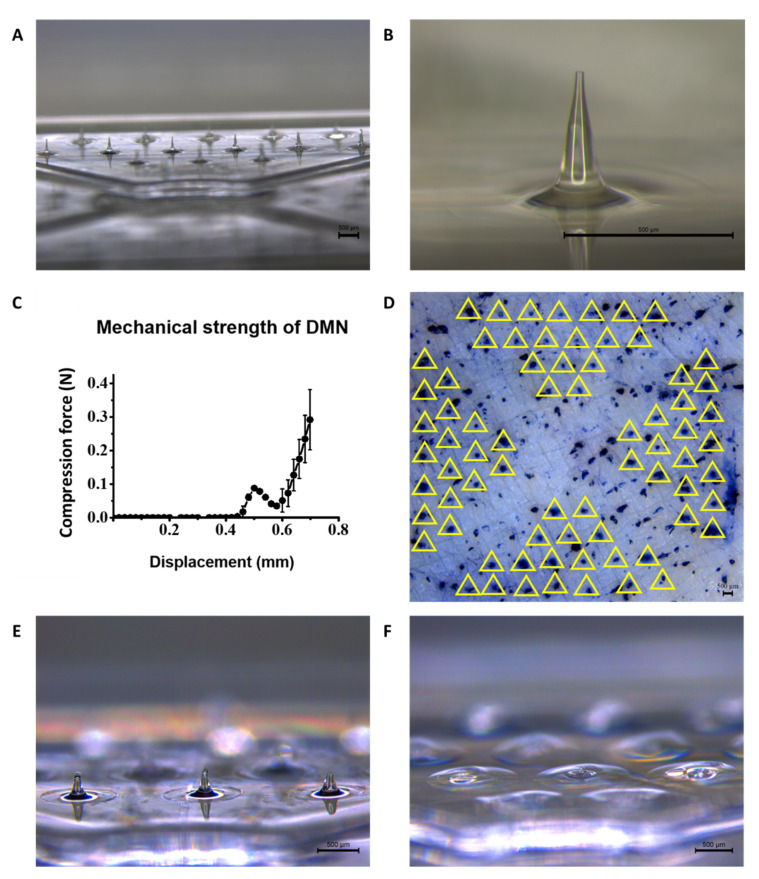
(**A**) Microscopic images of DMNs array fabricated on the disk and (**B**) single DMN. (**C**) Fracture force evaluation of single DMN fabricated on the disk. (**D**) Porcine skin was applied by DMNs fabricated on the disk using thumb force and stained with trypan blue to ascertain the skin penetration, and the penetrations were highlighted by the yellow triangles. (**E**) After thumb force application on the porcine skin without serum infusion, the DMNs on the disk were completely undissolved after 1 min and (**F**) completely dissolved when applied with serum infusion. The black bars represent 500 µm.

The DMNs fabricated on the disk with diagonal grooves are shown in Figure 2A. To ensure the morphological uniformity of each DMN on the disk, the physical properties of each DMN, especially the height and tip diameter, were assessed using microscopic images. As shown in Figure 2B, the DMNs array fabricated on the disk has a uniform ‘Eiffel tower’-like shape, and a single DMN image represents the smooth surface of DMN with the physical size. The average height and tip diameter of DMNs were 379.5 ± 12.7 μm and 28.0 ± 9.4 μm (n = 4, mean ± SEM), respectively.

To verify whether the mechanical properties of the DMNs are suitable for penetrating the skin to create micro-channels, fracture force evaluation of the single DMN and skin penetration analysis were performed. As shown in Figure 2C, the force analyzer probe starts to descend from a displacement of 0 mm and first contacts the DMN tip at the displacement approximate to 0.4 mm. After the contact, the force detected gradually increases until the displacement, where DMN fracture occurs. After the fracture of DMN, the detected force drastically decreases, then gradually increases again until the probe displacement of 0.7 mm. The fracture force of a single DMN was 0.088 ± 0.004 N, representing sufficient mechanical strength for skin penetration [23]. To validate the skin penetration by the DMNs and the dissolution of DMNs, the disks were applied to porcine skin using thumb force. Staining with trypan blue solution was performed after detachment of the disks for visualization. As shown in Figure 2D, the perforated skin channels formed by the DMNs array were fully stained with trypan blue solution, and the puncture pattern exactly matched the pattern of 72 DMNs fabricated on the disk. The results indicated that the thumb force application of the DMCS successfully penetrated each DMNs array into the porcine skin.

The serum-induced DMNs dissolution rate was compared to that of DMNs without serum. After 1 min of application of the disk without serum infusion, a considerable amount of DMNs remained, and the average height of the remaining DMNs was 279.7 ± 23.6 μm. However, in the case of a 1 min application of the disk with serum infusion, the DMNs were completely dissolved. The results indicated that embedded DMNs in the micro-channels formed in the porcine skin may have been drastically dissolved by the infused serum, thereby shortening the application time without waiting for the dissolution of DMN, thus enhancing user compliance.

### 3.3. Evaluation of Transdermal Serum Delivery Aided by DMCS

**Figure 3:**
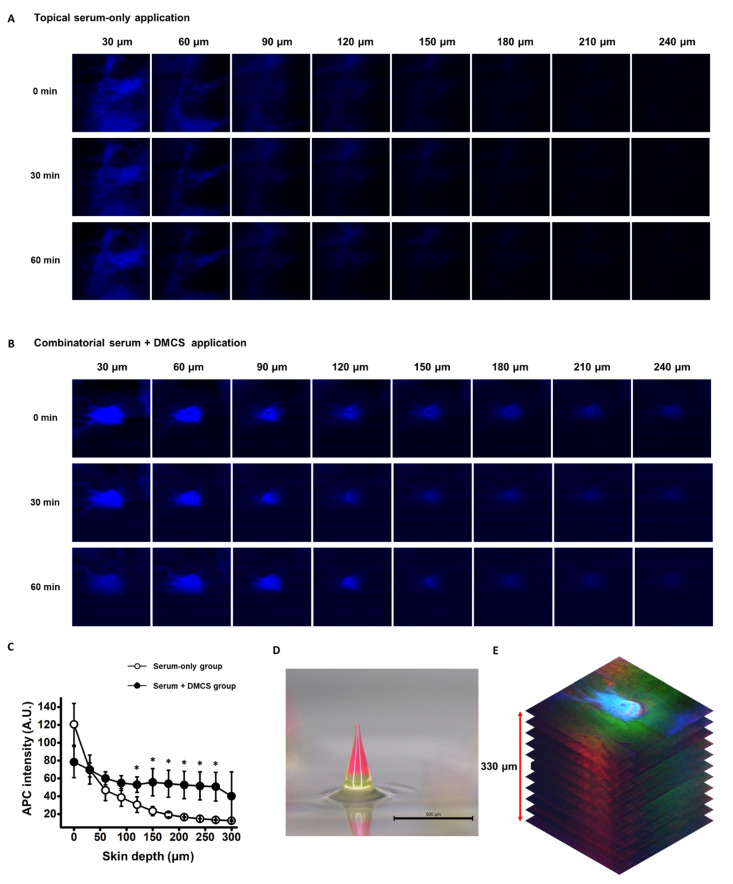
(**A**) Cross-sectional images of serum-delivered porcine skin with respective depths at 0, 30, and 60 min after the topical-serum-only application and (**B**) combinatorial application of serum and DMCS. (**C**) Detected APC intensities, which represent the serum delivery through the porcine skin at the respective depths at 0 min after the topical-serum-only application and combinatorial application of serum and DMCS. Statistical significance was evaluated by Student’s *t*-test, and the value of * *p* < 0.05 was statistically significant. (**D**) DMN fabricated on the disk loading calcein on the base layer and Rho B on the upper layer. (**E**) Stacked merged images of APC detection representing the serum delivery, and detection of calcein and Rho B delivered from the dissolved DMNs 0 min after the combinatorial application of serum and DMCS. Red arrows represent the length of 330 μm.

To enhance serum delivery to the deeper layer of the skin, serum delivery to the micro-channels with greater depth and diffusion of the serum through the skin tissue must be induced. After penetration of the surface of the porcine skin by DMNs, serum delivery to deeper layers of porcine skin via micro-channels was investigated. Since serum with APC (1%, *w/v*) was labeled blue, the porcine skin layers to which serum was delivered were detected as blue, as shown in Figure 3A.

The efficacy of transdermal serum delivery was compared between the serum-only application and the combinatorial application of DMCS and serum (Figure 3A,B). Although the intensities of APC gradually decreased as the depth of the skin increased in both methods, the degrees of reduction in APC intensities were different for the two methods. The APC-detectable range of skin depth with the naked eye ended at the 180 μm depth of porcine skin in the topical-serum-only applied porcine skin (Figure 3A). In contrast, the blue color was still detectable with the naked eye up to 240 μm depth of porcine skin when treated with the combinatorial method (Figure 3B). The blue colors forming the circles, which represent the cross-sectional shape of the micro-channels, gradually lose their intensity as the depth increases. This implied that the serum appropriately filled the micro-channels formed in the skin, and the serum could have been delivered to the deeper layers of the skin tissue via micro-channels compared to the topical serum application method.

APC intensities corresponding to depths of porcine skin from 0 μm to 300 μm with an interval of 30 μm were quantified, as shown in Figure 3C. At the layers corresponding to the depth range from 120 μm to 270 μm, APC intensities were significantly higher with the combinatorial application of serum and DMCS than with the serum-only application method. These results indicated that more serum could have been delivered to the deeper layers of porcine skin. Despite the lower intensity of APC at the 0 μm depth, signifying that a lower amount of serum was applied to the skin surface in the combinatorial application method, delivery of more serum to a deeper layer was achieved. This also indicated that the serum was more effectively delivered to a deeper layer via micro-channels.

The entire transdermal delivery phase of the dissolved DMNs and serum to the skin layers was observed in the combinatorial application. As shown in Figure 3D, DMNs were loaded with fluorescent dyes to observe the delivery of dissolved DMNs after serum application on the skin surface. The base layer of the DMN was labeled with calcein, and the upper layer was labeled with Rho B; therefore, porcine skin layers delivered with dissolved DMN were detected with green and red colors. APC-, calcein-, and Rho-B-detected images in the same layer of porcine skin were merged and stacked to form the image shown in Figure 3E. As shown in Figure 3E, the peripheral region of the pore on the porcine skin surface, which corresponds to a porcine skin layer depth of 0 μm, was detected with calcein, which is a green color. This indicates that the base layer containing calcein was not completely inserted into the skin. However, Rho B, which is detected in red, was detected in deeper layers, indicating that despite the incomplete insertion of DMNs, micro-channel formation and diffusion of Rho B from DMNs to the skin tissue were enabled. Based on these results, effective transdermal delivery of cosmetic active compounds, such as niacinamide, adenosine, and ascorbic acid, can be achieved by loading them onto the upper layer of the DMNs. Therefore, synergizing the delivery of serum and active compounds from dissolved DMNs can be further studied to enhance the cosmetic effects of the DMCS system.

### 3.4. Clinical Assessment: Skin Hydration Evaluation

**Figure 4:**
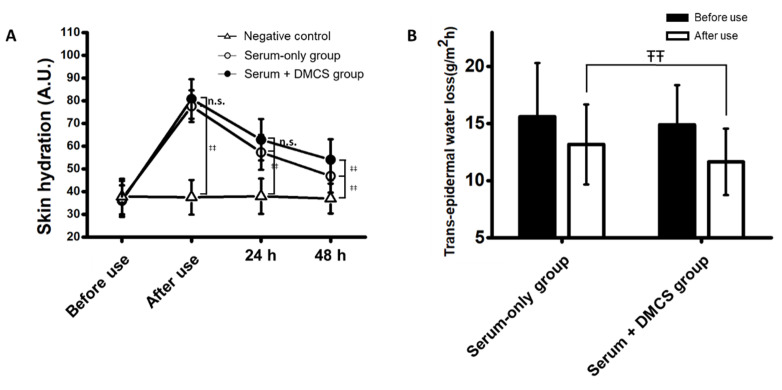
Clinical skin hydration analysis of serum delivery using the combinatorial application of serum with DMCS. (**A**) Change of skin hydration before use, after use, 24, and 48 h after use. (**B**) Trans-epidermal water loss changes of skin before use and after use. ‡‡: *p* < 0.017 according to Friedman test, post hoc Wilcoxon signed rank test with Bonferroni correction. ŦŦ: *p* < 0.05 according to ranked ANCOVA. n.s. means not significant.

Clinical efficacy was determined in the two groups, including the serum-only application group and the combinatorial application group, treated with DMCS and serum. The clinical efficacy for skin hydration in both groups was evaluated to compare the degrees of infiltration of niacinamide and adenosine, which are known to improve skin hydration. Skin hydration was determined by assessing moisture retention and trans-epidermal water loss, which are good indicators of the integrity of the skin’s ability to retain moisture. The improvement in moisture retention in both groups showed a similar trend (Figure 4A). Compared to the negative control, which was not treated with serum or DMCS, the moisture retention value commonly surged after use and showed significant improvement in skin hydration at every time point in both treatment groups. However, significant improvement in moisture retention was observed at 48 h after use in the combinatorial group compared to the serum-only application group. In addition, the trans-epidermal water loss significantly decreased after use in the combinatorial group compared to that in the serum-only application group (Figure 4B). The results indicated the superiority of the function enabling moisture retention and transdermal delivery of active compounds in the combinatorial application method compared with serum-only application.

### 3.5. Clinical Assessment: Evaluation of the Depigmenting Effect on Hyperpigmented Spots

**Figure 5:**
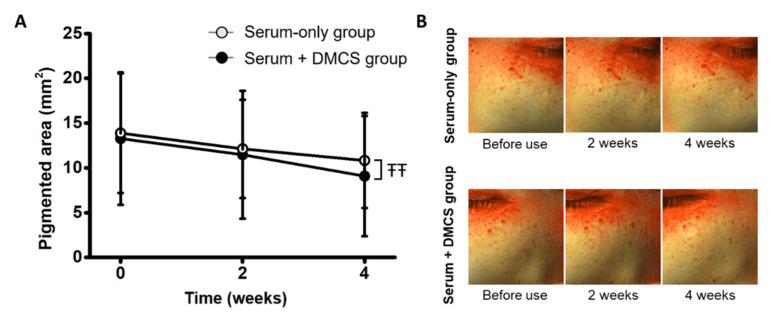
(**A**) Decrease in the pigmented areas before use, and at 2- and 4-week use. (**B**) Images of the pigmented areas and improvements of subjects. ŦŦ: *p* < 0.05 according to ranked ANCOVA.

The clinical efficacy for depigmentation on the skin was evaluated, as niacinamide and adenosine contained in the serum are known to suppress melanogenesis and melanosome transfer [37,38]. In the serum-only application group, serum was applied twice every day; in the combinatorial group, serum was applied twice every day, and a combinatorial application aided by DMCS was performed twice a week. The pigmented areas were assessed before use and at 2- and 4-week use. The hyperpigmented area showed a similar decreasing trend in both groups. The combinatorial application group showed a 13.57% and 12.70% decrease at 2- and 4-week use, respectively, and the serum-only application group showed a 12.70% and 21.00% decrease at 2- and 4-week use, respectively (Figure 5A). A significant depigmentation effect was observed 4 weeks after use, implying that active compound permeation was improved when combined with the micro-channel effect of DMCS. Furthermore, combinatorial application for more than 4 weeks will further depigment the skin, showing more significant performance compared to serum-only use. In addition, images of the hyperpigmented area produced by Antera 3D showed that the hyperpigmented areas gradually diminished after treatment, and no significant difference was found between the two groups.

### 3.6. Clinical Assessment: Evaluation of Skin Wrinkles through Three-Dimensional Visualization

**Figure 6:**
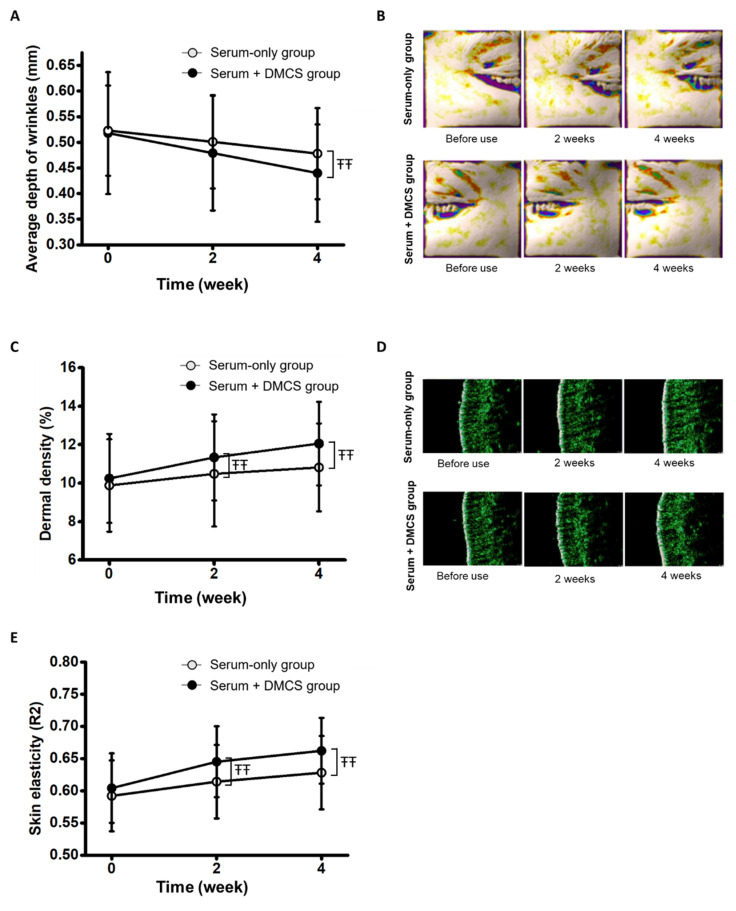
(**A**) Decrease in the average wrinkle depths of serum-only application group and combinatorial application group at before use, 2-, and 4-week use. (**B**) Three-dimensional visualized images of skin wrinkles in serum-only application group and combinatorial application group. (**C**) Dermal density improvement of serum-only application group and combinatorial application group at before use, 2-, and 4-week use. (**D**) Ultrasonographic images representing dermal density of serum-only application group and combinatorial application group. (**E**) Skin elasticity improvement of serum-only application group and combinatorial application group at before use, 2-, and 4-week use. ŦŦ: *p* < 0.05 according to ranked ANCOVA.

Functional compounds, such as niacinamide and adenosine, in the serum have been previously investigated to improve skin wrinkles [25,39]. The clinical efficacy in skin wrinkle improvement was assessed by measuring the average wrinkle depths of the crow’s feet region in both groups before use and at 2- and 4-week use. In both groups, the average wrinkle depth of the crow’s feet region decreased significantly. The average wrinkle depth of the combinatorial application group decreased by 7.53% and 15.06% at 2- and 4-week use, respectively, and that of the serum-only application group decreased by 4.21% and 8.60% at 2- and 4-week use, respectively (Figure 6A). After two weeks of use, no significant differences were found between the two groups. However, the combinatorial application group showed significantly greater wrinkle depth improvement than the serum-only application group at 4-week use. The results indicated that continuous combinatorial application and serum application for 4 weeks significantly improved wrinkles, and as the application time increased, the depth difference between the two groups increased. In addition, the gradual increase in differences between average wrinkle depths implies that longer term combinatorial use will induce more remarkable skin wrinkle enhancement. Furthermore, the function of niacinamide, which triggers a significant increase in collagen, would have been leveraged by the more substantial and more deeply delivering mechanism through micro-channels by combinatorial application [39]. As shown in Figure 6B, 3D skin wrinkle images processed by Antera 3D CS showed a gradual reduction in wrinkles in both groups after 2 weeks and 4 weeks of use. However, the skin wrinkles in the combinatorial treatment group were significantly improved compared to those in the serum-only application group (Figure 6B). Considering the collagen genesis induced during the wound healing process after micro-channeling, inserting DMNs may have contributed to the average skin wrinkle improvement [25]. Therefore, average skin wrinkle depths before use and at 2- and 4-week use were compared between the blank DMNs application group and the combinatorial application group (Figure S1). Average wrinkle depths were decreased by 2.43% and 5.96% at 2 and 4 weeks after blank DMNs application, respectively. Although the average wrinkle depth improvement was not as remarkable as serum-only and combinatorial applications, the small improvement is possibly attributed to the collagen genesis during wound healing after blank DMNs application.

### 3.7. Clinical Assessment: Dermal Density Assessment

As some of the clinical effects of niacinamide and adenosine contained in the serum are collagen genesis and dermal density increment, the serum used in the study was anticipated to improve dermal density [25,39]. Therefore, the clinical efficacy for dermal density improvement after use was assessed by quantitative analysis of dermal density (Figure 6C). Dermal densities in the serum-only application group and combinatorial application group significantly increased after 2- and 4-week use. However, the differences in increase between the two groups at 2 and 4 weeks of use were significant, and the value difference increased from 2 weeks to 4 weeks. The results indicated that using serum may improve dermal density, and combinatorial usage of DMCS with serum significantly leverages the dermal density increase effect of serum. Additionally, the increasing tendency of dermal density differences between the two groups shows the possibility of increasing the dermal density difference between the two groups after longer term use. Therefore, clinical investigations to verify more substantial performance after longer term combinatorial use are required. Ultrasonographic images of the skin showed that dermal density increased in both the serum-only application group and the combinatorial application group (Figure 6D). Considering the skin bulging effect observed in the 4-week-use image of the combinatorial application group, a relatively greater skin tightening effect of serum to the skin via micro-channels than that in the topical-serum-only application can be inferred. In addition, the dermal density improvement effect from the micro-channeling was investigated by comparing clinical dermal density at 2- and 4-week use of respective blank DMNs application and combinatorial application (Figure S2). Although no dermal density improvement was shown after the 2-week use of blank DMNs, the 4-week use of blank DMNs elicited dermal density improvement. Meager improvement of dermal density compared to the serum-only and combinatorial application groups may have been induced by the collagen genesis during wound healing after blank DMNs insertion.

### 3.8. Clinical Assessment: Skin Elasticity Assessment

Previously, niacinamide and adenosine contained in the serum were shown to increase skin elasticity, and the serum used in this study was expected to show clinical efficacy in improving skin elasticity [25,40]. The clinical assessment of skin elasticity was performed before use and at 2- and 4-week use. Skin elasticity was significantly increased in both the serum-only and combinatorial application groups after 2- and 4-week use. However, skin elasticity improvement was significantly different according to the application method, whereby the combinatorial application group showed a 6.79% and 9.60% increase at 2- and 4-week use, respectively, and the serum-only application group showed a 3.72% and 6.08% decrease at 2- and 4-week use, respectively (Figure 6E). The results indicated that the combinatorial application of serum and DMCS may induce a greater improvement in skin elasticity than serum-only application. The longer the maintenance of the applications, the larger the gap between the two groups, and thereby, the better skin elasticity improvement with the longer term use of combinatorial application would be elicited. Furthermore, the delivery of niacinamide and adenosine, which triggers collagen synthesis via micro-channels, may have led to the proliferation of collagen in the deeper layers of the skin, thereby significantly improving skin elasticity [39,40]. In addition, pure contribution to the skin elasticity improvement by micro-channeling was investigated by comparing skin elasticity after 2- and 4-week use of respective blank DMNs and combinatorial applications (Figure S3). Blank DMNs application elicited a 4.09% and 4.74% increase in skin elasticity at 2- and 4-week use, respectively, which significantly improved compared to before use. Considering better skin elasticity improvement shown in the combinatorial application compared to the serum-only and DMNs-only applications, the two application methods may have created synergistic effects to induce significantly greater improvement in skin elasticity.

### 3.9. Clinical Assessment: Skin Pore Number and Area Measurement

**Figure 7:**
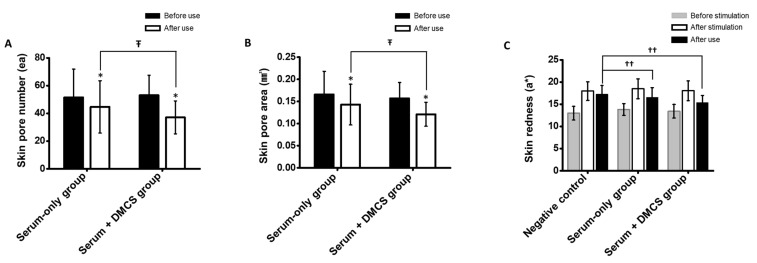
(**A**) Decrease in the average skin pore number and (**B**) average skin pore area of serum-only application group and combinatorial application group before and after use. (**C**) Skin redness before stimulation, after stimulation, and after soothing in the negative control group, serum-only application group, and combinatorial serum and DMCS application group. *: *p* < 0.05 according to paired *t*-test, Ŧ: *p* < 0.05 according to repeated-measures ANOVA, and ††: *p* < 0.05 according to one-way ANOVA.

The average skin pore number and area were evaluated, as niacinamide in the serum was previously investigated to reduce skin pores [39]. The average value reductions in skin pore number and skin pore area after use were evaluated in the serum-only application group and the combinatorial application group. As shown in Figure 7A,B, the average values of skin pore number and area were significantly reduced after use when compared to before use in both groups. However, there were significant differences in the degree of reduction in the average skin pore number and area between the serum-only application group and the combinatorial application group. The results indicated that a greater amount of niacinamide could have been delivered to the skin, which led to better shrinkage of skin pores, followed by skin pore removal and skin pore area reduction compared to the topical application. In addition, the rich delivery of functional compounds contained in the LFS and moisture through micro-channels may have induced closure of the skin pores.

### 3.10. Clinical Assessment: Evaluation of Skin Soothing Effect after External Stimulation

Since Lactobacillus loaded in LFS has been previously shown to reduce local skin inflammation and ameliorate skin barrier recovery [41], the skin soothing effects were evaluated after use. Changes in skin redness after stimulation in the negative control group without any treatment, serum-only application group, and combinatorial application group were assessed to evaluate the skin soothing effect. As shown in Figure 7C, the skin redness values of both groups increased after stimulation and decreased to different degrees. After stimulation, skin redness improved by 3.90%, 10.39%, and 14.84% in the negative control, serum-only application group, and combinatorial application group, respectively. Compared with the negative control, skin irritation was significantly ameliorated by topical-serum-only application and combinatorial application of serum and DMCS. Although an improved skin soothing effect was observed with the combinatorial application, it was not significant. This can be inferred from the micro-channel effect, which may merely inflict stimulation of the skin. Therefore, the improved soothing effects induced by the better delivery of functional compounds and Lactobacillus through micro-channels may have been offset. However, the skin soothing effect of the combinatorial application was at least equal to or beyond that of the serum-only application.

### 3.11. Clinical Assessment: Skin Irritation and Sensitization Assessment

During the clinical assessment of serum-only application and combinatorial serum and DMCS application groups, no abnormal skin reactions, including erythema, edema, and scale, were observed. In addition, no subjective irritations, including itching, tingling, burning, stiffness, and prickling, were reported by all testers who underwent self-observation.

## 4. Conclusions

This study indicates that the novel serum delivery method through the combinatorial application of topical serum and DMCS will improve the current skincare system. Topical serum application with DMNs embedded on the skin surface enhanced its otherwise poor delivery efficacy to the deep layer of the skin. In addition, the problems of low encapsulation capacity of active compounds due to the small volume of cosmetic DMNs and incomplete delivery of the active compounds due to poor insertion could be resolved by combining DMNs and topical serum applications. The successful findings of DMCS suggest the future direction of cosmetic technology, which is serum delivery via micro-channels. Furthermore, the combinatorial application of liquid formulations and DMNs is a promising candidate for achieving optimal drug delivery to the skin in the case of local diseases. It may deliver drugs more efficiently to the skin in atopic dermatitis and psoriasis via micro-channels for effective treatment.

## Data Availability

Not applicable.

## Supplementary Materials

The following supporting information can be downloaded at: https://www.mdpi.com/article/10.3390/pharmaceutics14122804/s1, Figure S1. Decrease in the average wrinkle depths of DMN-only application group and combinatorial application group before use, and at 2- and 4-week use. ŦŦ: *p* < 0.05 according to repeated ANOVA. Figure S2. Improvement in the skin elasticity of DMN-only application group and combinatorial application group before use, and at 2- and 4-week use. ŦŦ: *p* < 0.05 according to repeated ANOVA. Figure S3. Improvement in the dermal density of DMN-only application group and combinatorial application group before use, and at 2- and 4-week use. ŦŦ: *p* < 0.05 according to repeated ANOVA.
