# Supplementary material for: Enhanced Micro-Channeling System via Dissolving Microneedle to Improve Transdermal Serum Delivery for Various Clinical Skincare Treatments
Source: Pharmaceutics. 2022 Dec 14;14(12):2804. doi: 10.3390/pharmaceutics14122804 (PMC9781352; doi:10.3390/pharmaceutics14122804)
Supplement: Supplementary file 1 [file pharmaceutics-14-02804-s001.zip › pharmaceutics-1981707-supplementary.pdf]

Enhanced micro-channeling system via dissolving microneedle to improve transdermal serum delivery for various clinical skincare treatments

Jeeho Sim[1], SeongDae Gong[1,2], Geonwoo Kang[1,2], Mingyu Jang[2], Huisuk Yang[2], Jaesung Park[3], Youngchan Kim[3], Hyunkyu Lee[1,2], Hyunji Jung[1,2], Youseong Kim[1], Chansol Jeon[1,2], Hyeri Ahn[1], Minkyung Kim[1], Jaibyung Choi[1], Ho Lee[4,5,6] and Hyungil Jung [1,2,*]

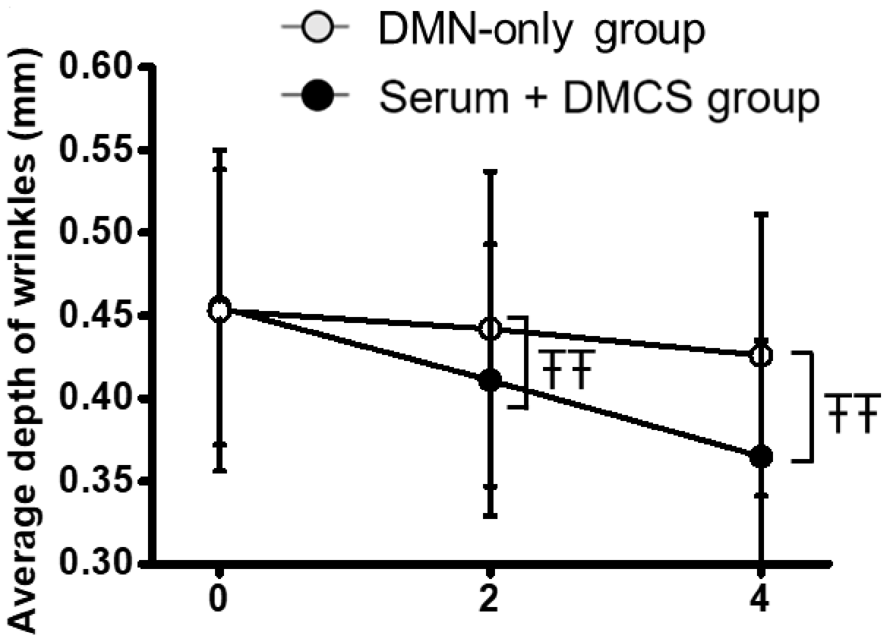

Figure S1. Decrease in the average wrinkle depths of DMN-only application group and combinatorial application group before use, 2-, and 4-week use. ŦŦ: $p < 0.05$ by repeated ANOVA.

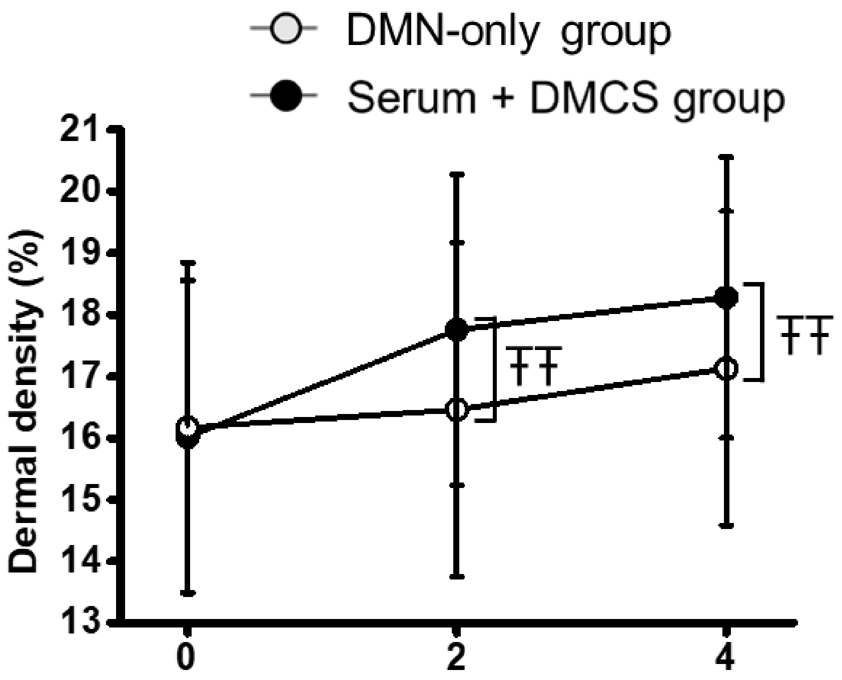

Figure S2. Improvement in the dermal density of DMN-only application group and combinatorial application group before use, 2-, and 4-week use. ŦŦ: $p < 0.05$ by repeated ANOVA

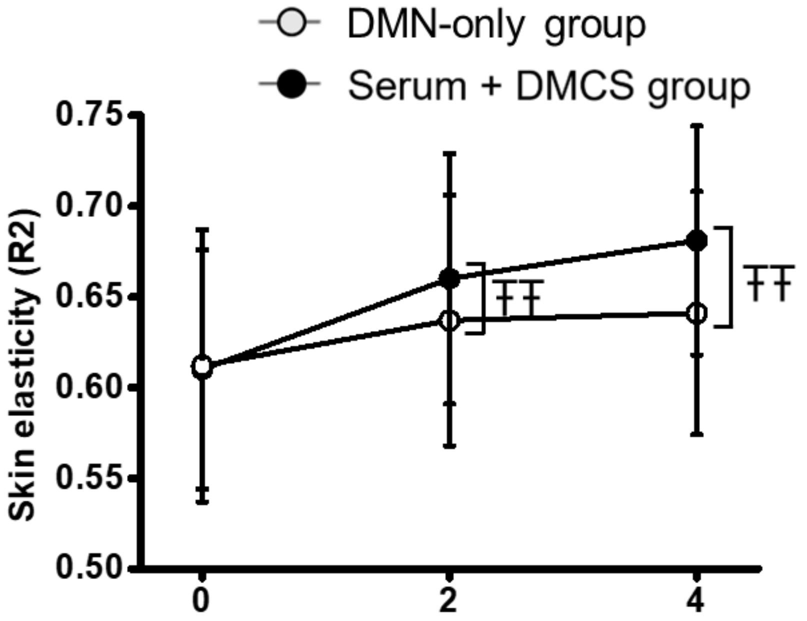

Figure S3. Improvement in the skin elasticity of DMN-only application group and combinatorial application group before use, 2-, and 4-week use. ŦŦ: $p < 0.05$ by repeated ANOVA.
